# Comparison of Survival Rates After a Combination of Local Treatment and Systemic Therapy vs Systemic Therapy Alone for Treatment of Stage IV Non–Small Cell Lung Cancer

**DOI:** 10.1001/jamanetworkopen.2019.9702

**Published:** 2019-08-21

**Authors:** Johannes Uhlig, Meaghan Dendy Case, Justin D. Blasberg, Daniel J. Boffa, Anne Chiang, Scott N. Gettinger, Hyun S. Kim

**Affiliations:** 1 Department of Diagnostic and Interventional Radiology, University Medical Center Göttingen, Göttingen, Germany; 2 Section of Interventional Radiology, Department of Radiology and Biomedical Imaging, Yale School of Medicine, New Haven, Connecticut; 3 Section of Thoracic Surgery, Department of Surgery, Yale New Haven Hospital, Yale University, New Haven, Connecticut; 4 Yale Cancer Center, Yale School of Medicine, New Haven, Connecticut; 5 Section of Medical Oncology, Department of Medicine, Yale School of Medicine, New Haven, Connecticut

## Abstract

**Importance:**

As many as 55% of patients with non–small cell lung cancer (NSCLC) present with stage IV disease at diagnosis. Although systemic therapy is the cornerstone for treatment of these patients, growing evidence suggests that local treatment of the primary tumor site may improve survival.

**Objective:**

To assess whether addition of local treatment for primary tumor site in stage IV NSCLC provides a survival benefit over systemic therapy alone.

**Design, Setting, and Participants:**

In this comparative effectiveness research study, the National Cancer Database (NCDB; 2018 version) was retrospectively queried from January 1, 2010, through December 31, 2015, for patients with a histopathologic diagnosis of stage IV NSCLC. Exclusion criteria were being younger than 18 years and missing information on tumor characteristics and follow-up data. Data were analyzed from November 1, 2018, through January 1, 2019.

**Exposures:**

Treatment groups were stratified as (1) surgical resection plus systemic therapy; (2) external beam radiotherapy (EBRT) or thermal ablation (TA; including cryosurgery and radiofrequency ablation) plus systemic therapy; and (3) systemic therapy alone.

**Main Outcomes and Measures:**

Overall survival was compared between treatment groups using multivariable Cox proportional hazards regression models and after propensity score matching. Subgroup analyses were planned a priori according to patient and tumor characteristics.

**Results:**

A total of 34 887 patients met inclusion criteria (19 002 male [54.5%]; median age, 68 years [interquartile range, 60-75 years]), among whom 835 underwent surgical resection plus systemic therapy; 9539, EBRT/TA plus systemic therapy; and 24 513, systemic therapy alone. Demographic and cancer-specific factors were associated with treatment allocation with a higher likelihood of surgical resection for oligometastatic NSCLC. After multivariable adjustment, surgical resection was associated with superior overall survival compared with EBRT/TA or systemic therapy alone (hazard ratio [HR] for EBRT/TA, 0.62; 95% CI, 0.57-0.67; *P* < .001; HR for systemic therapy alone, 0.59; 95% CI, 0.55-0.64; *P* < .001). Treatment with EBRT/TA demonstrated superior overall survival compared with systemic therapy alone (HR, 0.95; 95% CI, 0.93-0.98; *P* = .002). Interaction analyses identified heterogeneous associations with treatment; the EBRT/TA survival benefit was especially pronounced in stage IV squamous cell carcinoma with limited T and N category disease and oligometastases (HR, 0.68; 95% CI, 0.57-0.80; *P* < .001), with overall survival rates of 60.4% vs 45.4% at 1 year, 32.6% vs 19.2% at 2 years, and 20.2% vs 10.6% at 3 years for combination therapy vs systemic therapy alone.

**Conclusions and Relevance:**

In stage IV NSCLC, surgical resection or EBRT/TA of the primary tumor site may provide survival benefits in addition to systemic therapy alone in selected patients. Specifically, EBRT/TA may be considered as a treatment option in select patients who are ineligible for surgical resection.

## Key Points

**Question:**

Is there a survival benefit to combining local treatment and systemic therapy for stage IV non–small cell lung cancer?

**Findings:**

In this comparative effectiveness research study, overall survival of patients with stage IV non–small cell lung cancer was superior for combination of systemic therapy with surgical resection or external beam radiotherapy/thermal ablation of the primary tumor site compared with systemic therapy alone. Effectiveness of external beam radiotherapy/thermal ablation varied with histologic and other tumor characteristics.

**Meaning:**

In stage IV non–small cell lung cancer, surgical resection or external beam radiotherapy/thermal ablation of the primary tumor site may provide survival benefits in addition to systemic therapy alone for selected patients.

## Teaser

This comparative effectiveness research study assesses whether the addition of local treatment of the primary tumor site in stage IV non–small cell lung cancer (NSCLC) provides a survival benefit compared with systemic therapy alone.

## Introduction

Lung cancer remains the most common cause of cancer mortality worldwide.^1^ Non–small cell lung cancer (NSCLC) has a tendency to disseminate, with as many as 55% of patients presenting with stage IV disease.^2^ Systemic therapy has served as the cornerstone of treatment for stage IV lung cancer. Unfortunately, despite numerous innovations in targeted therapy and immunotherapy, most patients with stage IV lung cancer will die within 5 years of diagnosis.^3,4^ As a result, a novel approach to advanced lung cancer is needed desperately.

For the past several decades, physicians have recognized that a subset of patients with stage IV lung cancer whose dissemination is limited to 5 or fewer sites of disease (oligometastatic) appeared to have a more favorable outcome.^5^ In fact, highly selected patients with oligometastatic disease have been offered local therapy in hopes that eliminating all sites of disease would improve survival.^6,7,8^ Local therapy in the form of external beam radiotherapy (EBRT), local surgical resection, and thermal ablation (TA) have all been offered to highly selected patients with oligometastatic lung cancer to palliate a symptom or in an attempt to prolong survival.^9^ A recent randomized, prospective study of 74 patients with oligometastatic NSCLC^10^ identified superior progression-free survival with local control after hypofractionated radiotherapy or surgical resection and radiotherapy compared with systemic therapy alone, suggesting an important application of local treatment options for patients with metastatic disease. In an attempt to better characterize the use of local therapy in stage IV lung cancer in the United States and to estimate the effects of local therapy in this population, the treatment and outcomes of patients with stage IV lung cancer in the National Cancer Database (NCDB) were evaluated.

## Methods

The institutional review board of Yale University gave prior approval for this retrospective study on deidentified data and waived written informed consent. The presented study is compliant with the Health Insurance Portability and Accountability Act and followed the Strengthening the Reporting of Observational Studies in Epidemiology (STROBE) reporting guidelines. Following recommendations by Berger et al,^11^ the research question and analytic plan were specified a priori and remained unchanged.

The NCDB is jointly sponsored by the Commission on Cancer of the American College of Surgeons and the American Cancer Society.^12^ The NCDB contains approximately 34 million records from more than 1500 Commission on Cancer–accredited hospitals in the United States. Approximately 70% of annual US cancer cases are included. Commission on Cancer accreditation requires an annual follow-up rate of at least 90% for included patients within the first 5 years.

### Study Inclusion and Exclusion

The 2018 version of the NCDB was queried for adult patients diagnosed with histopathologically confirmed stage IV NSCLC (including large cell carcinoma, adenocarcinoma, and squamous cell carcinoma), according to the seventh edition of American Joint Committee on Cancer’s *Cancer Staging Manual*, from January 1, 2010, through December 31, 2015. Three cohorts were evaluated: (1) patients treated with surgical resection and systemic therapy; (2) patients receiving EBRT/TA with systemic therapy; and (3) patients receiving systemic therapy alone. The primary objective of this study was to assess the overall survival benefit of local therapy (surgical resection and/or EBRT/TA) in addition to systemic therapy for stage IV lung cancer compared with systemic therapy alone.

Exclusion criteria were being younger than 18 years and missing data (including TNM categories, survival status, follow-up time, metastatic spread pattern, or tumor diameter). Patients receiving treatment of metastatic tumor sites (ie, surgical resection of metastases or radiotherapy to metastatic sites) were excluded as well because the NCDB provided limited details for these cases.

### Variables

Because the NCDB classifies pharmacologic treatments as chemotherapy or immune therapy, and because this definition has changed during the study period, we summarized both strata as systemic therapy. Single- and multiple-agent systemic therapies were included. Surgical resection was defined as treatment by wedge resection, lobectomy, or pneumonectomy. External beam radiotherapy was defined as treatment with beam-based radiotherapy of the lung. To compare different EBRT protocols, the biological equivalent dose was calculated for each patient using the linear quadratic model with an α/β ratio of 10.^13^ Thermal ablation was defined as treatment with cryosurgery or laser ablation (NCDB surgical code 12) or radiofrequency ablation (NCDB surgical code 15 for “tissue destruction not otherwise specified”). T and N categories were classified according to clinical and pathologic assessment, whichever was rated higher. Timing of systemic therapy administration was available for patients receiving surgical resection only. Because the NCDB provided limited information on the extent of local treatments for metastatic sites, these patients were excluded from analyses as outlined above.

Patient-level variables evaluated included age, sex, race, insurance status, median household income according to zip code, proportion of residents with educational attainment of less than a high school diploma, comorbidities measured by Charlson-Deyo Comorbidity Index (stratified by the NCDB as 0, 1, 2, and ≥3), and duration from lung cancer diagnosis to the start of treatment. Hospital-level variables included facility type (academic vs nonacademic based on status of Commission on Cancer accreditation) and region. Tumor-level variables included year of cancer diagnosis, tumor diameter, histologic finding, location, pulmonary tumor spread, and the presence of metastasis (brain, bone, liver, or lung; available only for patients diagnosed after 2010). The number of metastatic sites was calculated from the number of lung, brain, liver, and bone metastases. For the scope of this study, oligometastatic NSCLC was defined as up to 1 distant metastasis to the lung, brain, liver, or bone. Given that only patients with stage IV NSCLC were included in our study, we assumed that in cases with no metastases reported in the NCDB, distant metastases were present to organ sites other than the lung, brain, liver, or bone (eg, adrenal glands).

Nodal status was coded using information from histopathologic finding and/or clinical manifestations of nodal involvement. Tumor histologic findings were stratified according to *International Classification of Disease–Oncology, Third Edition*, codes; tumor location was classified as lower, middle, or upper lobe or other location; and pulmonary tumor spread was classified as singular or multiple lung cancer nodules in the same or different lobe of the affected hemithorax.

### Statistical Analysis

Data were analyzed from November 1, 2018, through January 1, 2019. We compared continuous variables using the Wilcoxon rank sum test and categorical variables using the χ^2^ test. Median follow-up time was estimated using the reverse Kaplan-Meier method.

Owing to the small number of patients treated by surgical resection in certain subgroups, statistical modeling was conducted in 2 steps. First, overall survival comparing surgical resection with EBRT/TA and systemic therapy was conducted using Cox proportional hazards regression models in the full cohort, accounting for potential confounders in multivariable analyses and reporting hazard ratios (HRs) and associated 95% CIs. A robust SE estimate was used in the multivariable Cox model.

Second, we implemented a propensity score–matching procedure to compare EBRT/TA and systemic therapy alone after adjusting for potential confounders. A multivariable logistic regression model was used to determine which variables were associated with treatment with EBRT/TA. Variables with multivariable *P* < .05 were included to calculate the propensity score, and patients receiving EBRT/TA and systemic therapy were 1:1 matched. For propensity score matching, a greedy (nearest-neighbor) approach was chosen with maximum propensity score differences of ±1%. Success of the propensity score matching was tested via calculation of standardized mean difference for every matching variable. A priori planned subgroup analyses were conducted in the matched cohort to evaluate heterogeneous treatment effects according to patient and tumor characteristics.

All analyses were performed using R, version 3.4.3 (R Core Development Team), and RStudio, version 1.1.414 (R Studio Inc). An α level of .05 was chosen to indicate statistical significance. All provided *P* values are 2-sided.

## Results

A total of 34 887 patients fulfilled the inclusion criteria, of whom 19 002 were male (54.5%) and 15 885 were female (45.5%). Median age was 68 years (interquartile range [IQR], 60-75 years). Eight hundred thirty-five patients (2.4%) were treated by surgical resection; 9539 (27.3%), EBRT/TA; and 24 513 (70.3%), systemic therapy alone. The median biological equivalent dose of patients receiving EBRT was 46.9 Gy (IQR, 39.0-70.1 Gy) during a median course of 20 fractions (IQR, 10-31 fractions). Thermal ablation was used to treat 69 patients, of whom 39 received additional pulmonary EBRT.

### Treatment Allocation Factors

Patients treated by surgical resection or EBRT/TA as well as systemic therapy were more likely to be young white men with private insurance compared with those treated with systemic therapy alone (Table). In general, the delay between cancer diagnosis and treatment was shorter for patients undergoing systemic therapy and surgical resection or EBRT/TA (median, 16.0 months [IQR, 0.0-39.0 months] and 24.0 months [IQR, 12.0-38.0 months], respectively, vs 29.0 months [IQR, 18.0-46.0 months] for systemic therapy alone). Patients with adenocarcinoma were most likely to be treated with surgical resection (709 of 835 [84.9%]), whereas EBRT/TA was commonly used to treat squamous cell carcinoma (3814 of 9539 [40.0%]). Surgical resection was more common in patients with N0 to N1 category disease (506 of 835 [60.6%]), in cases of local tumor spread to the same lobe (116 of 835 [13.9%]) or a different lobe (164 of 835 [19.6%]), and in cases of limited systemic metastases (495 of 835 patients with ≥1 metastatic site reported [59.3%]) and M1a status (572 of 835 [68.5%]). Systemic therapy plus EBRT/TA and systemic therapy alone were more commonly used in patients with limited local spread but multiple systemic metastases (6686 of 9539 patients receiving EBRT/TA [70.1%] and 16 993 of 24 513 receiving systemic therapy [81.6%] with ≥2 metastatic sites reported). Geographic variability was evident across the United States, with the highest surgical resection rates reported in Middle Atlantic states (144 of 835 [17.2%]) and highest EBRT/TA rates reported in states of the East South Central region (1101 of 9539 [11.5%]) (eFigure in the Supplement).

**Table.** Baseline Characteristics of Included Patients

Variable	Patient Treatment Group^a^				*P* Value^b^
	All (n = 34 887)	Surgical Resection Plus Systemic Therapy (n = 835)	EBRT/TA Plus Systemic Therapy (n = 9539)	Systemic Therapy Alone (n = 24 513)	
Age, median (IQR), y	68 (60-75)	67 (59-73)	65 (57-73)	68 (61-75)	<.001
Sex					
Male	19 002 (54.5)	384 (46.0)	5587 (58.6)	13 031 (53.2)	<.001
Female	15 885 (45.5)	451 (54.0)	3952 (41.4)	11 482 (46.8)	
Race					
Black	4160 (11.9)	77 (9.2)	1242 (13.0)	2841 (11.6)	<.001
White	29 165 (83.6)	719 (86.1)	7964 (83.5)	20 482 (83.6)	
Other	1562 (4.5)	39 (4.7)	333 (3.5)	1190 (4.9)	
Insurance status					
Private	10 373 (29.7)	311 (37.2)	3032 (31.8)	7030 (28.7)	<.001
Medicare	19 942 (57.2)	462 (55.3)	4875 (51.1)	14 605 (59.6)	
Medicaid	2436 (7.0)	34 (4.1)	881 (9.2)	1521 (6.2)	
Government	419 (1.2)	13 (1.6)	161 (1.7)	245 (1.0)	
None or unknown	1717 (4.9)	15 (1.8)	590 (6.2)	1112 (4.5)	
Charlson-Deyo Comorbidity Index					
0	22 142 (63.5)	493 (59.0)	5947 (62.3)	15 702 (64.1)	.001
1	9079 (26.0)	251 (30.1)	2605 (27.3)	6223 (25.4)	
2	2693 (7.7)	63 (7.5)	731 (7.7)	1899 (7.7)	
≥3	973 (2.8)	28 (3.4)	256 (2.7)	689 (2.8)	
Time from diagnosis to treatment, median (IQR), mo	28.0 (16.0-44.0)	16.0 (0.0-39.0)	24.0 (12.0-38.0)	29.0 (18.0-46.0)	<.001
Histologic finding					
Adenocarcinoma	24 521 (70.3)	709 (84.9)	5419 (56.8)	18 393 (75.0)	<.001
Large cell carcinoma	972 (2.8)	14 (1.7)	306 (3.2)	652 (2.7)	
Squamous cell carcinoma	9394 (26.9)	112 (13.4)	3814 (40.0)	5468 (22.3)	
Separate tumor nodules					
None	22 616 (64.8)	426 (51.0)	6893 (72.3)	15 297 (62.4)	<.001
Ipsilateral different lobes	4151 (11.9)	164 (19.6)	970 (10.2)	3017 (12.3)	
Ipsilateral same and different lobe	5008 (14.4)	129 (15.4)	919 (9.6)	3960 (16.2)	
Ipsilateral same lobe	3112 (8.9)	116 (13.9)	757 (7.9)	2239 (9.1)	
Tumor location					
Lower lobe	9330 (26.7)	274 (32.8)	2037 (21.4)	7019 (28.6)	<.001
Middle lobe	1426 (4.1)	49 (5.9)	311 (3.3)	1066 (4.3)	
Other location	4719 (13.5)	128 (15.3)	1403 (14.7)	3188 (13.0)	
Upper lobe	19 412 (55.6)	384 (46.0)	5788 (60.7)	13 240 (54.0)	
Tumor grade					
I	1085 (3.1)	115 (13.8)	190 (2.0)	780 (3.2)	<.001
II	4824 (13.8)	287 (34.4)	1386 (14.5)	3151 (12.9)	
III	9723 (27.9)	294 (35.2)	3325 (34.9)	6104 (24.9)	
IV	273 (0.8)	12 (1.4)	80 (0.8)	181 (0.7)	
Unknown	18 982 (54.4)	127 (15.2)	4558 (47.8)	14 297 (58.3)	
Tumor size, median (IQR), mm	43.0 (28.0-63.0)	26.0 (16.0-42.0)	53.0 (36.0-74.0)	40.0 (27.0-59.0)	<.001
T category					
T1	5165 (14.8)	132 (15.8)	870 (9.1)	4163 (17.0)	<.001
T2	10 536 (30.2)	195 (23.4)	2603 (27.3)	7738 (31.6)	
T3	7630 (21.9)	216 (25.9)	2505 (26.3)	4909 (20.0)	
T4	11 556 (33.1)	292 (35.0)	3561 (37.3)	7703 (31.4)	
N category					
N0	7591 (21.8)	395 (47.3)	1715 (18.0)	5481 (22.4)	<.001
N1	3208 (9.2)	111 (13.3)	844 (8.8)	2253 (9.2)	
N2	15 786 (45.2)	257 (30.8)	4652 (48.8)	10 877 (44.4)	
N3	8302 (23.8)	72 (8.6)	2328 (24.4)	5902 (24.1)	
M category					
M1a	16 199 (46.4)	572 (68.5)	3723 (39.0)	11 904 (48.6)	<.001
M1b	18 688 (53.6)	263 (31.5)	5816 (61.0)	12 609 (51.4)	
Brain metastases					
Yes	3268 (9.4)	29 (3.5)	1745 (18.3)	1494 (6.1)	<.001
No	31 619 (90.6)	806 (96.5)	7794 (81.7)	23 019 (93.9)	
Skeletal metastases					
Bone metastasis	11 201 (32.1)	131 (15.7)	3003 (31.5)	8067 (32.9)	<.001
No bone metastasis	23 686 (67.9)	704 (84.3)	6536 (68.5)	16 446 (67.1)	
Liver metastases					
Yes	5606 (16.1)	49 (5.9)	1042 (10.9)	4515 (18.4)	<.001
No	29 281 (83.9)	786 (94.1)	8497 (89.1)	19 998 (81.6)	
Lung metastases					
Discontinuous or distant	11 711 (33.6)	349 (41.8)	2702 (28.3)	8660 (35.3)	<.001
None	23 176 (66.4)	486 (58.2)	6837 (71.7)	15 853 (64.7)	
No. of metastatic sites					
0	10 713 (30.7)	340 (40.7)	2853 (29.9)	7520 (30.7)	<.001
1	17 925 (51.4)	440 (52.7)	5224 (54.8)	12 261 (50.0)	
2	5022 (14.4)	47 (5.6)	1166 (12.2)	3809 (15.5)	
3	1091 (3.1)	8 (1.0)	248 (2.6)	835 (3.4)	
4	136 (0.4)	0	48 (0.5)	88 (0.4)	
Year of diagnosis					
2010	6225 (17.8)	187 (22.4)	1745 (18.3)	4293 (17.5)	.002
2011	6402 (18.4)	172 (20.6)	1758 (18.4)	4472 (18.2)	
2012	6838 (19.6)	132 (15.8)	1873 (19.6)	4833 (19.7)	
2013	7615 (21.8)	170 (20.4)	2038 (21.4)	5407 (22.1)	
2014	7807 (22.4)	174 (20.8)	2125 (22.3)	5508 (22.5)	
Treatment facility type					
Academic/research program	10 533 (30.2)	323 (38.7)	2538 (26.6)	7672 (31.3)	<.001
Other	24 354 (69.8)	512 (61.3)	7001 (73.4)	16 841 (68.7)	
Region of treatment facility location					
East North Central	6888 (19.7)	143 (17.1)	1952 (20.5)	4793 (19.6)	<.001
East South Central	3071 (8.8)	60 (7.2)	1101 (11.5)	1910 (7.8)	
Middle Atlantic	5032 (14.4)	144 (17.2)	1181 (12.4)	3707 (15.1)	
Mountain	1182 (3.4)	30 (3.6)	282 (3.0)	870 (3.5)	
New England	1980 (5.7)	51 (6.1)	451 (4.7)	1478 (6.0)	
Pacific	3170 (9.1)	83 (9.9)	737 (7.7)	2350 (9.6)	
South Atlantic	7934 (22.7)	194 (23.2)	2242 (23.5)	5498 (22.4)	
West North Central	2959 (8.5)	62 (7.4)	796 (8.3)	2101 (8.6)	
West South Central	2442 (7.0)	51 (6.1)	736 (7.7)	1655 (6.8)	
Suppressed for patients aged 0-39 y	229 (0.7)	17 (2.0)	61 (0.6)	151 (0.6)	

Abbreviations: EBRT, external beam radiotherapy; IQR, interquartile range; TA, thermal ablation.

^a^ Unless otherwise indicated, data are expressed as number (percentage) of patients. Percentages have been rounded and may not total 100.

^b^ Calculated using the Wilcoxon rank sum test for continuous variables and the χ^2^ test for categorical variables.

### Survival Analyses

**Figure 1.**
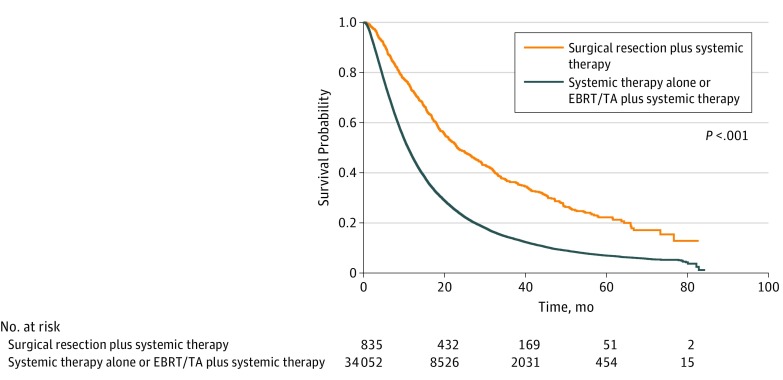
Overall Survival in Patients With Stage IV Non–Small Cell Lung Cancer Patients are stratified by treatment approach. EBRT/TA indicates external beam radiotherapy/thermal ablation.

Median follow-up was 39.4 months (IQR, 26.6-56.0 months). In the crude overall survival analysis (Figure 1), patients treated with surgical resection plus systemic therapy had superior survival compared with other treatments (HR vs EBRT/TA plus systemic therapy, 0.48; 95% CI, 0.44-0.52; *P* < .001; HR vs systemic therapy alone, 0.50; 95% CI, 0.47-0.54; *P* < .001). Crude overall survival rate at 1 year was 72.9% after surgical resection plus systemic therapy. These differences persisted after adjustment for confounders, such as preferential surgical resection in oligometastatic NSCLC; on multivariable analyses, superior overall survival was identified for surgical resection plus systemic therapy (HR vs EBRT/TA plus systemic therapy, 0.62; 95% CI, 0.57-0.67; *P* < .001; HR vs systemic therapy alone, 0.59; 95% CI, 0.55-0.64; *P* < .001) (eTable 1 in the Supplement). EBRT/TA plus systemic therapy demonstrated superior overall survival compared with systemic therapy alone (HR, 0.95; 95% CI, 0.93-0.98; *P* = .002).

### Overall Survival After EBRT/TA and Systemic Therapy

**Figure 2.**
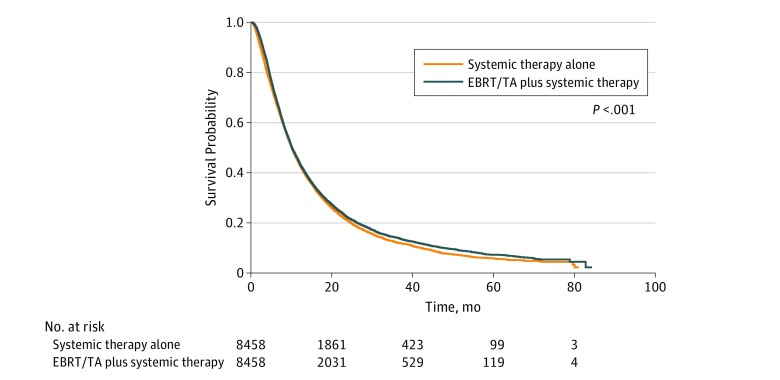
Overall Survival in Patients With Stage IV Non–Small Cell Lung Cancer After Accounting for Confounders via Propensity Score Matching Superior overall survival was identified for external beam radiotherapy/thermal ablation (EBRT/TA) vs systemic therapy alone (hazard ratio, 0.94; 95% CI, 0.91-0.97; *P* < .001).

For subgroup analyses and interaction testing, patients receiving EBRT/TA and systemic therapy underwent 1:1 propensity score matching. Propensity matching focused on balancing known confounders between both treatment groups, including the extent of local and systemic tumor spread. The matched cohort included 16 916 patients, yielding standardized mean differences of 0.1 or below (with the exception of longer time to treatment and smaller tumor diameter in the systemic therapy subgroup) (eTable 2 in the Supplement). In the propensity score–matched cohort, superior overall survival was identified for EBRT/TA plus systemic therapy vs systemic therapy alone (HR, 0.94; 95% CI, 0.91-0.97; *P* < .001) (Figure 2).

### Subgroup Analyses

**Figure 3.**
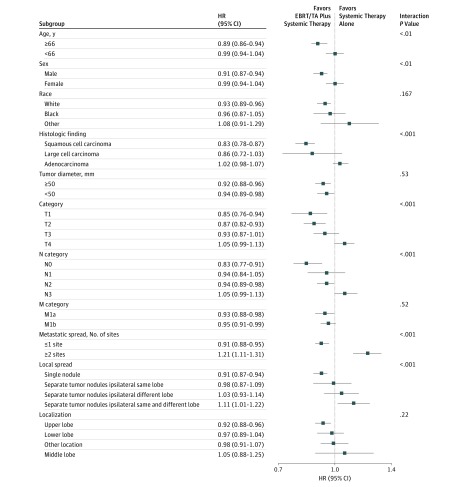
Overall Survival Subgroup Analyses of Patients Receiving External Beam Radiotherapy/Thermal Ablation (EBRT/TA) and Systemic Therapy vs Systemic Therapy Alone for Stage IV Non–Small Cell Lung Cancer Patients underwent 1:1 propensity score matching. Age and tumor diameter were dichotomized at the median. Metastatic spread was arbitrarily dichotomized.

Subgroup analyses were also performed to detect heterogeneous effects of treatment depending on patient demographics and cancer factors (Figure 3). Qualitative interactions were seen depending on cancer histologic finding (*P* < .001 for multivariable interaction), T category (*P* < .001 for multivariable interaction), N category (*P* < .001 for multivariable interaction), number of metastatic sites (*P* < .001 for multivariable interaction), and local cancer spread (*P* < .001 for multivariable interaction). Further quantitative interactions were evident for patient age and sex. Based on these interactions, subgroup analyses were conducted to describe the benefit of EBRT/TA and systemic therapy alone in selected patient cohorts.

### Patient Selection for EBRT/TA and Systemic Therapy Alone

**Figure 4.**
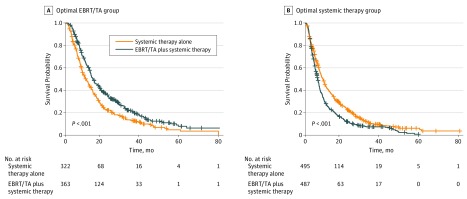
Subgroup Analyses Showing the Potential Benefit of External Beam Radiotherapy/Thermal Ablation (EBRT/TA) and Systemic Therapy vs Systemic Therapy Alone in Selected Populations Optimal EBRT/TA group consisted of patients with T1 to T2 category disease, N0 to N1 category disease, and oligometastatic squamous cell carcinoma. Optimal systemic therapy group consisted of patients with T3 to T4 category disease, N2 to N3 category disease, 2 or more distant metastases, and adenocarcinoma.

To further quantify the potential benefit of EBRT/TA in selected patients with stage IV NSCLC, a subgroup analysis from the matched cohort was conducted in patients with squamous cell carcinoma, T1 to T2 category disease, N0 to N1 category disease, and oligometastases. As shown in Figure 4, patients treated with EBRT/TA had a survival benefit compared with those receiving systemic therapy alone (HR, 0.68; 95% CI, 0.57-0.80; *P* < .001). Overall survival rates were 60.4% vs 45.4% at 1 year, 32.6% vs 19.2% at 2 years, and 20.2% vs 10.6% at 3 years for combination treatment vs systemic therapy alone. In contrast, patients with adenocarcinoma, T3 to T4 category disease, N2 to N3 category disease, and 2 or more distant metastatic sites had an inferior overall survival after EBRT/TA compared with patients undergoing systemic therapy alone (Figure 4) (HR, 1.39; 95% CI, 1.22-1.59; *P* < .001), with overall survival rates at 1 year of 41.4% vs 25.3%; at 2 years of 19.5% vs 10.6%; and at 3 years of 9.4% vs 7.4%.

## Discussion

Systemic therapy is considered the standard of care for stage IV NSCLC across a wide array of oncology treatment guidelines.^14,15,16^ The prospect of improving outcomes with a treatment management paradigm that includes locoregional therapy of the primary tumor site, whether surgical resection, EBRT, or TA, is promising, especially given initial results from phase 2 studies.^10,17^

Using the NCDB, this study demonstrates that the addition of a local treatment option for the primary tumor site combined with systemic therapy was associated with overall survival, even after accounting for potential confounders. Patients undergoing surgical resection of their primary cancer appeared to benefit the most, with superior overall survival rates of 72.9% at 1 year. Further, a survival benefit was evident for EBRT/TA compared with systemic therapy alone, although its clinical relevance remains unclear given the limited effect size. Nevertheless, subgroup analyses indicate that the survival benefit of EBRT/TA varied by patient and cancer-specific variables; in optimally selected patients presenting with squamous cell carcinoma and limited nodal disease as well as oligometastases, EBRT/TA yielded a clinically relevant overall survival benefit vs systemic therapy alone. In contrast, EBRT/TA treatment in patients with adenocarcinoma and extended local and distant spread was associated with unfavorable prognosis vs systemic therapy alone. Patient demographic and cancer factors were strongly associated with treatment allocation; for example, surgical resection was primarily used for patients with small, oligometastatic NSCLC. However, based on our results, patients beyond this selected cohort might benefit from surgical resection.

Several arguments support the use of localized therapy for the primary tumor site, even in stage IV NSCLC. For example, in patients with oligometastatic and less aggressive stage IV lung cancer, tumor progression is most commonly observed at the primary site of disease.^18^ Addressing the primary tumor with surgical resection, EBRT, or TA may therefore contribute to observed increases in overall survival. Avoiding early resistance to systemic therapy is likely an additional advantage that explains this observed benefit, because local tumor cell destruction aids in controlling disease before malignant cells can mutate and develop systemic therapy resistance.^19^ Abscopal effects should also be considered as an underlying mechanism for observed survival discrepancies, because increased immunogenicity of tumors after local treatment may improve the effects of systemic immunotherapy.^20^ An argument for early use of local therapy for metastatic sites is the linear progression model, whereby metastases can further disseminate secondary metastases.^21^ Local treatment of pulmonary metastases could thereby potentially decrease propagation of future disease.

Previous research supports our findings on the utility of local therapy in conjunction with systemic therapy for smaller, selected patient cohorts with limited metastases.^22^ Recently, Gomez et al^10^ and Iyengar et al^23^ have demonstrated the benefit of local consolidative therapy, defined as surgical resection or chemoradiotherapy, in patients with oligometastatic NSCLC. Palma and colleagues^17^ reported increased survival after EBRT for oligometastatic tumor of various sites, including lung tumors, vs standard of care. Several investigators^7,24,25,26,27^ have found similar results, with overall survival rates ranging from 32% at 18 months to 43% at 24 months. Conversely, Downey et al^28^ reported no improvement in survival for patients with a solitary synchronous NSCLC metastasis treated with surgical resection of all disease sites and systemic chemotherapy. In a recent phase 2 trial, Sutera et al^29^ reported a favorable complication profile for EBRT in oligometastatic lung cancer with acute grade 2 complications in only 7.5% of patients.

Several studies also underline the effectiveness of local tumor destruction. For example, Li et al^30^ investigated the efficacy of radiofrequency ablation after first-line chemotherapy in stages IIIb to IV NSCLC, reporting a median progression-free survival of 16 weeks. Lee et al^31^ studied the combination of chemotherapy and radiofrequency ablation vs chemotherapy alone in patients with stages III to IV NSCLC and found combination therapy to be superior, with a median survival of 42 and 29 months, respectively. Ni et al^32^ reported that oligoprogressive metastases in NSCLC may be another target for thermal ablation.

Very few studies have evaluated locoregional therapy in oligometastatic NSCLC with exclusive extracranial metastases. Prior work has shown that survival of patients with definitive treatment of a primary lung tumor and single brain metastasis can be similar to that for stage I NSCLC.^33^ However, these observations have not been validated for extracranial metastases in oligometastatic NSCLC. For example, Tanvetyanon et al^34^ completed a systematic review of patients with oligometastatic NSCLC with either synchronous or metachronous adrenal metastasis and found no survival advantage associated with surgical treatment of distant disease.

The present study supports a combined approach of local therapy in addition to systemic treatment for select patients with oligometastatic NSCLC. Prospective research should focus on combination treatment for this subgroup, including the benefits of TA, in patients with oligometastatic disease who cannot tolerate surgical resection or receive EBRT. Furthermore, the utility of this treatment for distant disease control should be evaluated. Of high interest are 2 active prospective studies: the UK Conventional Care vs Radioablation (Stereotactic Body Radiotherapy) for Extracranial Oligometastases (CORE) trial assessing stereotactic body radiotherapy before systemic chemotherapy and the NRG-LU002 trial evaluating stereotactic body radiotherapy after induction chemotherapy and before maintenance therapy.^35^ Another trial with recruitment planned for late 2019 aims to assess the benefit of surgical resection of the primary NSCLC site after immunotherapy, chemotherapy, and EBRT for oligometastatic lesions.^36^ Especially with the advent of immunotherapy for lung cancer treatment, the role of local therapies needs to be defined. Given that lung cancer immunotherapies were only recently commercially approved, our results might not represent the most recent therapeutic advances.

### Limitations

This study has several limitations that are mainly inherent to a large retrospective data set such as the NCDB. First, crucial information on lung function is missing that might have biased patient selection for the various local treatment options. However, confounding by lung function is unlikely to fully explain the observed survival differences in this study, particularly the large increase in overall survival for surgical resection. The NCDB does not provide details on specific systemic therapy protocols or targeted therapies. Differences in systemic therapy administration contribute to the survival benefit seen for surgical resection and EBRT/TA. No information was given regarding exact tumor localization and extent, which may have driven treatment decisions and could confound these results. The small number of patients limits some of the subgroup analyses and might impair the generalizability of our results. Although several metastatic sites were evaluated in the NCDB, details were not available on adrenal metastases. Excluding patients receiving surgical resection of metastatic sites, we were unable to assess potential benefits of the procedure. Further, the sequence of systemic therapy and EBRT/TA was not specified, which rendered discrimination between EBRT/TA as an initial treatment vs a treatment for local progressive disease impossible. Because the NCDB did not provide details on NSCLC mutational status, actionable mutations could have been unbalanced between treatment groups, which might in turn have biased our results. Although the NCDB includes approximately 70% of annually diagnosed cancer cases, these data may not be fully representative of the US population.

## Conclusions

This study found that in stage IV NSCLC, surgical resection or EBRT/TA of the primary tumor site in addition to systemic therapy may confer additional survival benefit compared with systemic therapy alone in select patients. The addition of EBRT/TA to systemic therapy may be considered a treatment option in select patients who are not eligible for surgical resection. The benefits of EBRT/TA in conjunction with systemic therapy vs systemic therapy alone are especially pronounced in select patients with squamous cell carcinoma, those with limited T and N category disease, and those with oligometastases. We believe future studies are warranted to assess the combination therapies and sequencing of systemic and local therapies in stage IV NSCLC.

## Notes

Supplement. **eFigure.** Proportion of Patients With Stage IV Lung Cancer Receiving Surgical Resection and EBRT/TA According to US Region **eTable 1.** Multivariable Cox Proportional Hazards Regression Model Predicting Overall Survival **eTable 2.** Characteristics of 1:1 Propensity Score–Matched Patients
