# Supplementary material for: Comparison of Survival Rates After a Combination of Local Treatment and Systemic Therapy vs Systemic Therapy Alone for Treatment of Stage IV Non–Small Cell Lung Cancer
Source: JAMA Netw Open. 2019 Aug 21;2(8):e199702. doi: 10.1001/jamanetworkopen.2019.9702 (PMC6707019; doi:10.1001/jamanetworkopen.2019.9702)
Supplement: Supplement. — eFigure. Proportion of Patients With Stage IV Lung Cancer Receiving Surgical Resection and EBRT/TA According to US Region eTable 1. Multivariable Cox Proportional Hazards Regression Model Predicting Overall Survival eTable 2. Characteristics of 1:1 Propensity Score–Matched Patients [file jamanetwopen-2-e199702-s001.pdf]

Supplementary Online Content

Uhlig J, Case MD, Blasberg JD, et al. Comparison of survival rates after a combination of local treatment and systemic therapy vs systemic therapy alone for treatment of stage IV non–small cell lung cancer. *JAMA Network Open.* 2019;2(8):e199702. doi:10.1001/jamanetworkopen.2019.9702

eFigure. Proportion of Patients With Stage IV Lung Cancer Receiving Surgical Resection and EBRT/TA According to US Region

eTable 1. Multivariable Cox Proportional Hazards Regression Model Predicting Overall Survival

eTable 2. Characteristics of 1:1 Propensity Score–Matched Patients

This supplementary material has been provided by the authors to give readers additional information about their work.

© 2019 Uhlig G et al. *JAMA Network Open*

eFigure. Proportion of Patients With Stage IV Lung Cancer Receiving Surgical Resection and EBRT/TA According to US Region

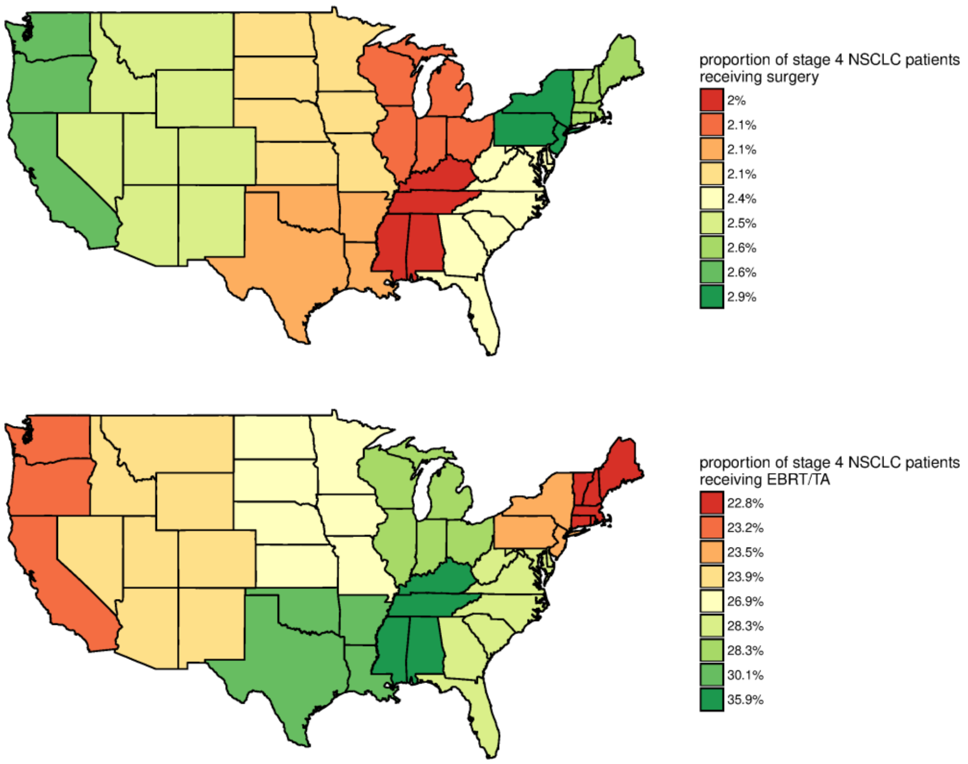

© 2019 Uhlig G et al. *JAMA Network Open*

eTable 1. Multivariable Cox Proportional Hazards Regression Model Predicting Overall Survival

**predictor**	**HR**	**lower.95.CI**	**upper.95.CI**	**pval**
treatment				
systemic therapy alone (reference)	1			
surgical resection	0.59	0.55	0.64	<0.001
EBRT/TA+systemic therapy	0.95	0.93	0.98	0.002
age [years]	1.01	1.01	1.01	<0.001
race				
African American (reference)	1			
Caucasian	1.01	0.97	1.05	0.594
others	0.7	0.66	0.75	<0.001
insurance status				
private insurance (reference)	1			
Medicare	1.08	1.04	1.12	<0.001
Medicaid	1.21	1.15	1.27	<0.001
govt. insurance	1.19	1.07	1.33	<0.001
not insured/unknown insurance	1.17	1.11	1.25	<0.001
Charlson comorbidity index				
0 (reference)	1			
1	1.16	1.13	1.2	<0.001
2	1.31	1.25	1.37	<0.001
>=3	1.48	1.37	1.6	<0.001
days to treatment	1	1	1	<0.001
histology				
adenocarcinoma	1			
large cell carcinoma	1.32	1.22	1.42	<0.001
squamous cell carcinoma	1.2	1.17	1.24	<0.001
size	1	1	1	<0.001
T-Status				
T1 (reference)	1			
T2	1.08	1.04	1.12	<0.001
T3	1.2	1.14	1.25	<0.001

© 2019 Uhlig G et al. *JAMA Network Open*

T4	1.23	1.18	1.29	<0.001
N-Status				
N0	1			
N1	1.12	1.07	1.17	<0.001
N2	1.24	1.2	1.28	<0.001
N3	1.3	1.25	1.35	<0.001
M-Status				
M1a	1			
M1b	1.01	0.98	1.04	0.432
tumor location				
lower lobe (reference)	1			
middle lobe	0.99	0.93	1.05	0.642
other location	1.06	1.02	1.11	0.004
upper lobe	1	0.97	1.03	0.955
tumor nodules				
singular nodule (reference)	1			
separate tumor nodules ipsilateral different lobe	0.9	0.86	0.94	<0.001
separate tumor nodules ipsilateral same and different lobe	0.9	0.86	0.94	<0.001
separate tumor nodules ipsilateral same lobe	0.96	0.92	1.01	0.091
liver metastases				
liver metastases present (reference)	1			
no liver metastasis	0.72	0.68	0.76	<0.001
lung metastases				
lung metastases present (reference)	1			
no lung metastasis	1.06	1.01	1.13	0.033
brain metastases				
brain metastases present (reference)	1			
no brain metastasis	0.85	0.8	0.9	<0.001
bone metastases				
bone metastases present (reference)	1			
no bone metastasis	0.82	0.78	0.87	<0.001
number of metastatic sites				
0 (or not detailed in NCDB; reference)	1			
1	0.95	0.89	1	0.047
2	1.06	0.95	1.17	0.311
3	1.03	0.87	1.21	0.759
4	NA			
tumor grading				
Grade I (reference)	1			
Grade II	1.05	0.98	1.13	0.176

© 2019 Uhlig G et al. *JAMA Network Open*

Grade III	1.21	1.13	1.29	<0.001
Grade IV	1.17	1	1.37	0.053
unknown grade	1.12	1.04	1.19	<0.001
facility type				
academic center (reference)	1			
non-academic center	1.15	1.12	1.18	<0.001
Year of diagnosis				
2014 (reference)	1			
2010	1.2	1.15	1.25	<0.001
2011	1.12	1.08	1.16	<0.001
2012	1.1	1.05	1.14	<0.001
2013	1.06	1.02	1.1	0.004

© 2019 Uhlig G et al. *JAMA Network Open*

eTable 2. Characteristics of 1:1 Propensity Score–Matched Patients			
	**Total**<br>**No. 16,916**	**EBRT/TA+ systemic therapy**<br>**No. 8,458**	**systemic theray alone**<br>**No. 8,458**
age	66.0 (58.0 - 73.0)	66.0 (58.0 - 73.0)	66.0 (58.0 - 73.0)
gender			
female	7,186 (42.5%)	3,597 (42.5%)	3,589 (42.4%)
male	9,730 (57.5%)	4,861 (57.5%)	4,869 (57.6%)
race			
African American	2,142 (12.7%)	1,100 (13.0%)	1,042 (12.3%)
Caucasian	14,089 (83.3%)	7,059 (83.5%)	7,030 (83.1%)
others	685 (4.0%)	299 (3.5%)	386 (4.6%)
insurance status			
private insurance	5,306 (31.4%)	2,613 (30.9%)	2,693 (31.8%)
Medicare	9,058 (53.5%)	4,491 (53.1%)	4,567 (54.0%)
Medicaid	1,372 (8.1%)	717 (8.5%)	655 (7.7%)
govt. insurance	228 (1.3%)	136 (1.6%)	92 (1.1%)
not insured/unknown insurance	952 (5.6%)	501 (5.9%)	451 (5.3%)
comorbidities [Charlson Score]			
0	10,540 (62.3%)	5,294 (62.6%)	5,246 (62.0%)
1	4,566 (27.0%)	2,272 (26.9%)	2,294 (27.1%)
2	1,353 (8.0%)	660 (7.8%)	693 (8.2%)
3	457 (2.7%)	232 (2.7%)	225 (2.7%)
time diagnosis to treatment	26.0 (14.0 - 40.0)	25.0 (13.0 - 40.0)	27.0 (16.0 - 41.0)
histology			
adenocarcinoma	10,021 (59.2%)	5,060 (59.8%)	4,961 (58.7%)
large cell carcinoma	549 (3.2%)	259 (3.1%)	290 (3.4%)
squamous cell carcinoma	6,346 (37.5%)	3,139 (37.1%)	3,207 (37.9%)
tumor nodules			
no separate tumor nodules	11,805 (69.8%)	5,921 (70.0%)	5,884 (69.6%)
separate tumor nodules ipsilateral different lobe	1,850 (10.9%)	920 (10.9%)	930 (11.0%)
separate tumor nodules ipsilateral same and different lobe	1,820 (10.8%)	903 (10.7%)	917 (10.8%)
separate tumor nodules ipsilateral same lobe	1,441 (8.5%)	714 (8.4%)	727 (8.6%)
tumor location			
lower lobe	3,896 (23.0%)	1,921 (22.7%)	1,975 (23.4%)
middle lobe	597 (3.5%)	299 (3.5%)	298 (3.5%)
other location	2,425 (14.3%)	1,224 (14.5%)	1,201 (14.2%)
upper lobe	9,998 (59.1%)	5,014 (59.3%)	4,984 (58.9%)
tumor grade			
Grade I	352 (2.1%)	182 (2.2%)	170 (2.0%)
Grade II	2,409 (14.2%)	1,211 (14.3%)	1,198 (14.2%)
Grade III	5,696 (33.7%)	2,805 (33.2%)	2,891 (34.2%)
Grade IV	150 (0.9%)	73 (0.9%)	77 (0.9%)
unknown grade	8,309 (49.1%)	4,187 (49.5%)	4,122 (48.7%)
tumor size [mm]	50.0 (33.0 - 70.0)	51.0 (35.0 - 70.0)	49.0 (32.0 - 70.0)
T status			
T1	1,685 (10.0%)	853 (10.1%)	832 (9.8%)
T2	4,909 (29.0%)	2,452 (29.0%)	2,457 (29.0%)
T3	4,230 (25.0%)	2,116 (25.0%)	2,114 (25.0%)
T4	6,092 (36.0%)	3,037 (35.9%)	3,055 (36.1%)
N status			
N0	3,105 (18.4%)	1,546 (18.3%)	1,559 (18.4%)
N1	1,506 (8.9%)	757 (9.0%)	749 (8.9%)
N2	8,102 (47.9%)	4,045 (47.8%)	4,057 (48.0%)

© 2019 Uhlig G et al. *JAMA Network Open*

N3	4,203 (24.8%)	2,110 (24.9%)	2,093 (24.7%)
M Status			
M1a	7,048 (41.7%)	3,523 (41.7%)	3,525 (41.7%)
M1b	9,868 (58.3%)	4,935 (58.3%)	4,933 (58.3%)
brain metastases			
brain metastasis	2,274 (13.4%)	1,124 (13.3%)	1,150 (13.6%)
no brain metastasis	14,642 (86.6%)	7,334 (86.7%)	7,308 (86.4%)
skeletal metastases			
bone metastasis	5,506 (32.5%)	2,737 (32.4%)	2,769 (32.7%)
no bone metastasis	11,410 (67.5%)	5,721 (67.6%)	5,689 (67.3%)
liver metastases			
liver metastasis	2,001 (11.8%)	1,005 (11.9%)	996 (11.8%)
no liver metastasis	14,915 (88.2%)	7,453 (88.1%)	7,462 (88.2%)
lung metastases			
discontinuous or distant metastases to the lung	5,114 (30.2%)	2,552 (30.2%)	2,562 (30.3%)
no lung metastasis	11,802 (69.8%)	5,906 (69.8%)	5,896 (69.7%)
number of metastatic sites			
0	5,423 (32.1%)	2,606 (30.8%)	2,817 (33.3%)
1	8,722 (51.6%)	4,592 (54.3%)	4,130 (48.8%)
2	2,221 (13.1%)	998 (11.8%)	1,223 (14.5%)
3	469 (2.8%)	218 (2.6%)	251 (3.0%)
4	81 (0.5%)	44 (0.5%)	37 (0.4%)
year of diagnosis			
2010	3,079 (18.2%)	1,551 (18.3%)	1,528 (18.1%)
2011	3,095 (18.3%)	1,548 (18.3%)	1,547 (18.3%)
2012	3,325 (19.7%)	1,638 (19.4%)	1,687 (19.9%)
2013	3,619 (21.4%)	1,830 (21.6%)	1,789 (21.2%)
2014	3,798 (22.5%)	1,891 (22.4%)	1,907 (22.5%)
treatment facility type			
academic/research program	4,610 (27.3%)	2,294 (27.1%)	2,316 (27.4%)
other treatment facility type	12,306 (72.7%)	6,164 (72.9%)	6,142 (72.6%)

© 2019 Uhlig G et al. *JAMA Network Open*
